# Establishment and characterization of oviductal organoids from farm and companion animals^†^

**DOI:** 10.1093/biolre/ioad030

**Published:** 2023-03-14

**Authors:** Edwina F Lawson, Arnab Ghosh, Victoria Blanch, Christopher G Grupen, Robert John Aitken, Rebecca Lim, Hannah R Drury, Mark A Baker, Zamira Gibb, Pradeep S Tanwar

**Affiliations:** Priority Research Centre for Reproductive Science, University of Newcastle, Callaghan, NSW, Australia; Global Centre for Gynaecological Diseases, University of Newcastle, Callaghan, NSW, Australia; School of Biomedical Sciences and Pharmacy, University of Newcastle, Callaghan, NSW, Australia; Hunter Medical Research Institute, Newcastle, NSW, Australia; Global Centre for Gynaecological Diseases, University of Newcastle, Callaghan, NSW, Australia; School of Biomedical Sciences and Pharmacy, University of Newcastle, Callaghan, NSW, Australia; Hunter Medical Research Institute, Newcastle, NSW, Australia; Sydney School of Veterinary Science, Faculty of Science, University of Sydney, Camden, NSW, Australia; Priority Research Centre for Reproductive Science, University of Newcastle, Callaghan, NSW, Australia; School of Biomedical Sciences and Pharmacy, University of Newcastle, Callaghan, NSW, Australia; Hunter Medical Research Institute, Newcastle, NSW, Australia; School of Biomedical Sciences and Pharmacy, University of Newcastle, Callaghan, NSW, Australia; Hunter Medical Research Institute, Newcastle, NSW, Australia; Priority Research Centre for Reproductive Science, University of Newcastle, Callaghan, NSW, Australia; Hunter Medical Research Institute, Newcastle, NSW, Australia; Priority Research Centre for Reproductive Science, University of Newcastle, Callaghan, NSW, Australia; Global Centre for Gynaecological Diseases, University of Newcastle, Callaghan, NSW, Australia; School of Biomedical Sciences and Pharmacy, University of Newcastle, Callaghan, NSW, Australia; Hunter Medical Research Institute, Newcastle, NSW, Australia

**Keywords:** oviduct, organoids, fertility, disease modeling, equine

## Abstract

Organoid technology has provided a unique opportunity to study early human development and decipher various steps involved in the pathogenesis of disease. The technology is already used in clinics to improve human patient outcomes. However, limited knowledge of the methodologies required to establish organoid culture systems in domestic animals has slowed the advancement and application of organoid technology in veterinary medicine. This is particularly true for the field of reproduction and the application of assisted reproductive technologies (ART). Here, we have developed a platform to grow oviductal organoids from five domestic species—bovine, porcine, equine, feline, and canine. The organoids were grown progressively from single cells derived from the enzymatic digestion of freshly collected infundibular/fimbrial samples. The addition of WNT, TGFβ, BMP*,* ROCK, and Notch signaling pathway activators or inhibitors to the organoid culture medium suggested remarkable conservation of the molecular signals involved in oviductal epithelial development and differentiation across species. The gross morphology of organoids from all the domestic species was initially similar. However, some differences in size, complexity, and growth rate were subsequently observed and described. After 21 days, well-defined and synchronized motile ciliated cells were observed in organoids. Histopathologically, oviductal organoids mimicked their respective native tissue. In summary, we have carried out a detailed cross-species comparison of oviductal organoids, which would be valuable in advancing our knowledge of oviduct physiology and, potentially, help in increasing the success of ART.

## Teaser

Organoids can be derived from the oviductal epithelium of bovine, feline, canine, equine, and porcine to advance assisted reproductive technologies in animals.

## Graphical abstract

**Figure:**
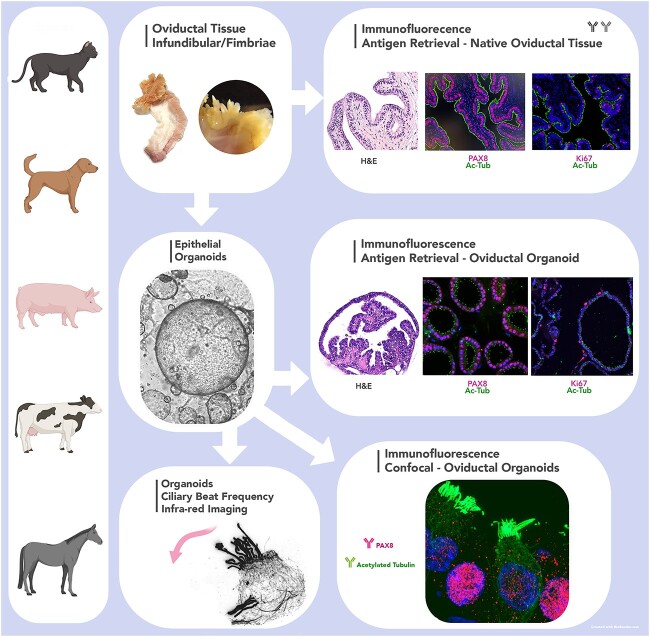

## Introduction

The oviduct is not solely a conduit that connects the ovary to the uterus, but a highly complex hormonally regulated organ, which is essential to sustain fertilization and early embryonic life [1]. Organoids are defined as 3D structures grown from stem cells and consisting of organ-specific cell types that self-organize through spatially restricted lineage [2]. Organoids have been instrumental in carrying out human and mouse research on the anatomically obscure oviduct. However, in domestic species, because oviductal physiology and fertilization strategies vary greatly between mammalian species, organoid technology has been limited.

Oviducts (fallopian tubes in primates) are paired tubular organs and consist of four functionally unique segments. The funnel-shaped infundibulum is proximal to the ovary, whose fimbrial branches pick up the newly ovulated oocyte [3–5]. The ampullary region is where sperm and egg meet, and fertilization begins. The ampulla connects to the isthmus, which is believed to play a role in the storage of spermatozoa before ovulation, as well as regulating the transport of cleaving embryos. Lastly is the proximately located utero-tubal junction [6], the region in which embryos remain before reaching the uterine lumen [7]. The lumen of the mammalian oviduct is lined by intricately folded pseudostratified epithelial cells containing interspersed secretory and ciliated cells [8]. Ciliated cells propel the oocyte by creating luminal currents through the continual synchronized beating of their cilia axoneme creating fluidic turbulence [4]. Whereas secretory cells produce the complex oviductal fluid, which provides the optimal microenvironment for sperm capacitation, fertilization, and early embryonic survival [9, 10].

Like many epithelia, the oviduct in mice and human contains a population of stem/progenitor cells responsible for epithelial regeneration and maintenance [11, 12]. These epithelial cells are regularly exposed to follicular fluid during each ovulation, which contains copious inflammatory cytokines and reactive oxygen species (ROS) [13, 14]. This cyclic exposure to cytokines/ROS in conjunction with ovarian hormones is suspected to cause tissue injury and DNA damage to the oviductal epithelium, especially to the epithelial cells residing at the distal end of the oviducts [13, 14]. Thus, the distal (fimbrial) oviductal epithelium of both mouse and human oviducts is shown to have the most abundant stemness activity, which is required for the regeneration of oviductal epithelium [11, 12, 15, 16]. Lineage tracing studies in mouse models have identified Pax8+ secretory cells as the oviductal stem/progenitor cells [11, 17]. Similar studies using primary oviductal epithelial (OE) cells have also identified Pax8+ secretory cells as stem/progenitor cells in human oviducts [12, 18]. These cells, in the presence of a supportive cell culture environment with required niche factors, can develop organoids that can be propagated in culture for more than a year [11, 12, 16]. It is currently unclear if similar stem/progenitor cells are present in the oviducts of other species, as most of the previous work is focused on the mouse and human oviducts.

In a clinical setting, patient-derived organoids are utilized to conduct in vitro drug efficacy testing for tailored treatments facilitating the use of personalized medicine in clinical practice [19]. Oviductal organoids composed of fully differentiated ciliated and secretory epithelial cells have been successfully generated from human [12, 20] and mouse-derived cells [16, 17]. While these systems have vastly improved the understanding of pathologies and the process of fertilization in each of these species, their applicability to other animals remains limited. As such, the aim of this study was to carry out a detailed cross-species comparison of OE organoid development.

## Material and methods

### Epithelial isolation and organoid culture

**Figure 1:**
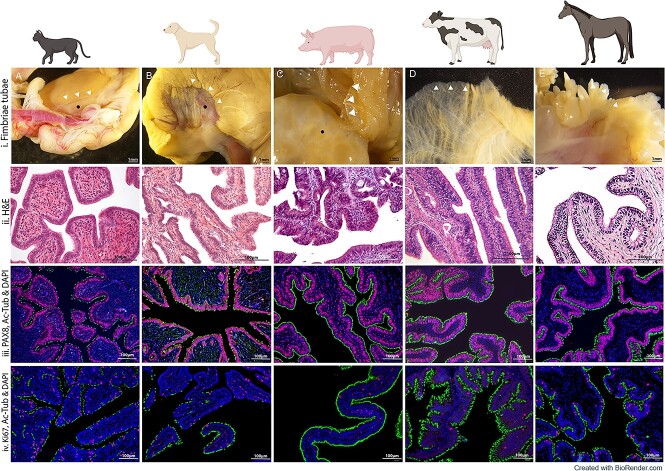
Characterization of feline (A), canine (B), porcine (C), bovine (D), and equine (E) native infundibular/fimbriae oviductal tissue. (i) Gross anatomical features of oviductal infundibular/fimbriae, including the ovary in feline, canine, and porcine images (black dot). The distal portions of the infundibulum branch to tightly engulf the ovary (white arrow) in feline and canine. In the porcine, bovine, and equine, the projections are not adhered to the ovary but freely interact with the ovarian structure. (ii) Histological staining with H&E details the infundibular epithelium, lined with a single layer of polarized columnar epithelial cells. In each species, a highly folded mucosa with crypts is evident. (iii) Immunofluorescence imaging shows a consistent expression of PAX8 (red) positive secretory epithelial cells, located mainly along the basal region of the epithelial cells. Additionally, AC-Tub (green) is depicted staining of the ciliated epithelial cells. In the cat and dog, AC-Tub positive ciliated cells were more irregularly spaced than those in porcine, bovine, and equine. DAPI (blue) staining shows the nuclei of cells. (iv) Immunostaining depicting positive staining for Ki67 (red). Mature ciliated cells are stained positive for AC-Tub (green), and cell nuclei are stained with DAPI (blue). Cells can be seen dispersed throughout the normal healthy oviductal epithelium.

Whole oviducts were obtained from adult porcine (*n* = 3), bovine (*n* = 2), equine (*n* = 4), canine (*n* = 5), and feline (*n* = 4) females. Tissue samples were collected either directly from the slaughterhouse or from discarded postsurgical hysterectomy material. All samples were collected, during summer from non-cycling juveniles, except for equine, which were obtained from cycling mares aged 2, 7, 10, and 12 years. No formal institutional animal ethics committee approval was required as this work was conducted using opportunistic sourcing of discarded tissues and organs. After collection, samples were then washed and transported on ice in a transportation medium (Dulbecco Modified Eagle Medium/Nutrient Mixture F-12 (DMEM-F12 cat# 12634-010) with 1% penicillin–streptomycin (Life Technologies, cat# 15070-063) and stored in at 4°C overnight in the same medium. Oviducts were washed three times with Dulbecco phosphate-buffered saline and 1% penicillin–streptomycin to remove the blood and unwanted debris. Using a stereomicroscope, the finger-like projections analogs to the fimbriae of human fallopian tubes in the most distal portion of the infundibulum were dissected for cell isolation (white arrowheads, Figure 1). Then multiple fringes were dissected from the whole infundibulum, and digested in Accumax (Innovative Cell Technologies, Inc., cat#AM105) enzyme for 2–3 h on a shaker at room temperature. Enzymatic digestion was stopped by adding “complete medium” comprising 5% fetal bovine serum (FBS), 1% Glutamax (Sigma), 1% HEPES, and 1% penicillin–streptomycin in Advanced DMEM-F12. The cell suspension was collected in a Falcon tube and centrifuged at 1500 rpm for 5 min to collect the epithelial cells. These epithelial cells were then propagated as a 2D culture in the same complete medium supplemented with human Epidermal Growth Factor (hEGF) (12 ng/ml, PeproTech), ROCK inhibitor (Y-27632; 10 mM; TOCRIS), and Primocin (0.2%) on a matrigel coated cell culture plate. Once 70% confluence had been achieved in 3–5 days, epithelial cells were detached by trypsinization and centrifuged at 1500 rpm for 5 min to collect the epithelial cells. Epithelial cells in fresh complete medium (without above-mentioned growth factors such as, hEGF, Y-27632, primocin) were then incubated for 2.5–3 h at 37°C, in a 5% CO_2_ incubator for differential attachment to enrich pure epithelial cells. In brief, during differential attachment, 80–90% stromal fibroblast and blood cells will attach to the cell culture plate during 2.5–3 h, whereas the epithelial cells take longer than 3 h to start attaching to the culture plate surface. Thus, this method can achieve 80–90% pure epithelial cells for the organoid culture. After epithelial cell enrichment, the cells were counted and resuspended in Advanced DMEM-F12 with 1% Glutamax (Sigma), 1% HEPES, and 1% penicillin–streptomycin, referred to as OE culture medium. Following this, the epithelial cells were mixed with matrigel and placed as a 50 μl drop in each well in a 24-well cell culture plate, with 25 000 cells in each 50 μl drop. The matrigel with cells was allowed to solidify by keeping the 24-well plate at 37°C for 20 min. Then each well was overlaid with 75% OE medium and 25% WNT3A–RSPO3–NOGGIN-conditioned medium (WRN-CM) supplemented with growth factors and signaling modulators comprising hEGF (12 ng/ml, PeproTech), B27 Supplement (2%, Gibco), ROCK inhibitor (Y-27632 dihydrochloride; 5 mM; TOCRIS), N-Acetyl-L-Cysteine (1.25 mM/ml, Sigma), Nicotinamide (10 mM/ml, Sigma), Primocin (0.2%, Invivogen), and A83-01 (0.5 mM, TOCRIS). For individual wells in the 24-well plate, we used 500 ml of the above-mentioned culture medium, applying the same conditions for all species. Medium for organoid culture was replenished every 3 days, and on day 21 the organoids were harvested for further processing.

L-WRN cell line expressing Wnt-3A, R-spondin3, and noggin was purchased from ATCC and WRN-CM was prepared from L-WRN cells by following a detailed published protocol [21]. Long-term culture was achieved by passaging a population of organoids routinely every 7–10 days. Manual pipetting was carried out to break the Matrigel dome gently. TrypLE (Thermo Fisher Scientific, Cat#12605010) was then added before incubating at 37°C for 5–7 min. Trypsinization was stopped by adding FBS before gentle pipetting to break up cell clumps. Cells were centrifuged at 1500 rpm for 5 min before resuspending in Matrigel [22]. A proportion of organoids were frozen in 50% FBS Media +10% DMSO and stored in liquid nitrogen.

### Immunofluorescence and hematoxylin and eosin

Oviductal tissues were fixed in 4% (w/v) paraformaldehyde at 4°C overnight before being processed for paraffin embedding. Organoids were fixed in 4% (w/v) paraformaldehyde for 1 h at room temperature. The paraffin-embedded blocks were then sectioned at 5 μm thickness. For IF and hematoxylin and eosin (H&E) staining, sections were deparaffinized followed by rehydration, and processed separately. For immunofluorescence (IF) staining, antigen retrieval was carried out on rehydrated sections by heating either 110°C (oviductal tissue) or 98°C (organoids) for 30 min in EDTA buffer (1 mM; pH 8.0). After blocking, sections were incubated with primary antibodies at 4°C overnight. The primary antibodies used were rabbit anti-PAX8 (1:1000; catalog number 10336-1-AP; Proteintech), mouse anti-Acetylated-Tubulin (AC-Tub) (1:1000; catalog number T7451, clone 6-11B-1, Sigma-Aldrich), rabbit anti-Ki67 (1:400; catalog number Ab15580, Abcam), and DAPI dilactate (Sigma-Aldrich D9564). The secondary antibodies were Alexa 488/594-conjugated anti-rabbit/mouse IgG (1:250; Jackson ImmunoResearch Labs).

### Microscopy and image acquisition

Initial gross oviductal images were captured using a Nikon SMZ25 stereoscope. During organoid culture, organoid development images were taken by JuLiTM Stage Real-Time Cell History Recorder (NanoEnTek) every 3 days after media replenishment. As the organoids diffusely distribute within the Matrigel dome, images were taken on multiple focal planes through each dome at these timepoints. Immunofluorescence and H&E images were acquired using Olympus DP80 CCD (charge-coupled device) and cellSens software (Olympus) on the afore mentioned sections. Confocal laser scanning was carried out on a Zeiss LSM 900-Airyscan-2 microscope with a ×63 objective using the software ZEN (blue edition) 3.0 and were reconstructed by sequences (*z*-stack).

### Quantification and statistical analysis

Organoid count and measurement analysis was carried out retrospectively on brightfield images using Fiji 9/ImageJ [23], counting consecutively those organoids that appeared in-focus across the planes. For each sample, 25 representative organoids were considered and measured at each time point for each species. To quantify the distinct changes in morphology, the principles of morphometrics were applied using perimeter to area ratio to measure the structural complexity [24, 25]. To assess whether organoid area differed according to time point, separate one-way analyses of variance were conducted with days (6, 12, 18, and 21) as independent variable, and organoid area as dependent variable. Tukey’s multiple comparisons tests were computed for pairwise differences in mean organoid areas among measurement days. For cell quantification, statistical analyses were conducted using SAS version 9.4 (SAS Institute Inc.) on the previously captured IF images, using Fiji 9/ImageJ to acquire cell count. Separate independent sample *t*-tests were conducted to examine differences in the percentages of PAX8, AC-Tub, and Ki67 found in each species. All tests were two-sided with a significance level of 0.05.

### Ciliated cell recordings

The Matrigel domes containing mature organoids (<21 days) were pipetted multiple times to expose apical side and transferred directly to a room temperature (22–25°C) recording chamber containing OE culture medium. A 22 mm diameter glass coverslip formed the base of the recording chamber, which had sloped edges to form a well in which the organoids were captured and secured in place using a platinum harp with strings. The recording chamber had an in-line for cell culture media and a suction electrode attached to a vacuum pump, to remove cell culture media [26]. The total volume of the well was 1 ml and a steady and continuous flow rate of two bath volumes per minute (i.e. 3 ml/min) containing OE culture medium was perfused. Cilia were visually recorded in real time under a Zeiss Axioskop microscope using infra-red differential interference contrast optics and a Zeiss AxioCam Mrm camera. From the equine and porcine organoid populations, cilia were readily visible and hence imaged using Zen Blue software (version 2.6). Real-time live ciliary motions were recorded from 12 regions of ciliated axonemes at 30-s intervals. The recording captured a synchronous and continual beating of motile cilia, which generated a coordinated and unidirectional flow in both species (Supp. 1). To confirm findings, repeat cilia recording sessions were carried out three times over a 5-month period.

## Results

### Gross and histological characterization of infundibulum from domestic animals

Healthy feline, canine, porcine, bovine, and equine oviducts were collected, and gross images of morphological infundibular characteristics of each species were obtained, as shown (Figure 1i). Despite the fimbriae/infundibulum serving the same function across the species, namely, to collect ovulated oocytes and transport them, apparent morphological differences were evident. Macroscopically, the infundibulum presents funnel-shaped irregular projections (white arrows) with branching fimbriae engulfing the ovary (Figure 1). In porcine, bovine, and equine, the fimbrial branches were more distinct and readily identifiable compared with feline and canine. Similar to mouse oviducts, the infundibulum adheres tightly to the ovary in the feline and canine oviducts (Figure 1iA and iB). However, porcine, equine, and bovine oviducts are more similar to human fallopian tubes where the infundibulum and its fimbrial folds branch more loosely around the ovary without being attached to the ovaries (Figure 1iC–iE). Histological analysis revealed similar histoarchitecture of native oviductal mucosa (Figure 1ii). The oviductal epithelium from each species comprised a single convoluted layer of columnar epithelial cells lining the lumen, creating both luminal projections and crypts of varying dimensions.

### Distribution of secretory and ciliated cells in oviducts

Immunofluorescence analysis of native fimbrial/infundibular epithelium was initially carried out. The secretory cell marker PAX8, which is localized in the nuclei of healthy OE cells, showed a strong consistent nuclear expression in all species examined (Figure 1iii and iv). Using DAPI as a nuclear counterstain, AC-Tub was used to identify the small hair-like protuberances of the ciliated cells by staining the acetylated microtubules found within centrioles and cilia. Notably, in the feline and canine, AC-Tub+ ciliated cells were consistently expressed but punctate and more irregularly spaced between secretory cells throughout the folds (Figure 1iiiA and iiiB). When comparing this patterning to that of the porcine, bovine, and equine (Figure 1iiiC–iiiE), it appears that the population of ciliated cells is more consistent. In these species, non-ciliated secretory cells can be seen more sparsely interspersed between the ciliated cells. Furthermore, Ki67, a cell proliferation marker [22], was observed dispersed throughout normal healthy oviductal epithelium (Figure 1iv). The presence of Ki67 and its distribution suggests some OE cells are actively proliferating.

### Establishment of oviductal organoid cultures

The most distal fringes of the fimbriae/infundibulum were collected from equine, porcine, bovine, canine, and feline oviducts. Using enzymatic digestion, single cells were isolated, embedded in Matrigel, and cultured in an organoid culture medium supplemented with required growth and niche factors (detailed in the materials and method section). Organoids progressively developed from single cells over the course of 21 days. The organoids were imaged every 3 days (Figure 2i), to record a timeline of the visual change occurring within the cultures, including morphological changes and size, as measured by perimeter (μm). Cultures were efficiently generated long term and maintained for several months. Organoids were also successfully grown from cells frozen in liquid nitrogen.

**Figure 2:**
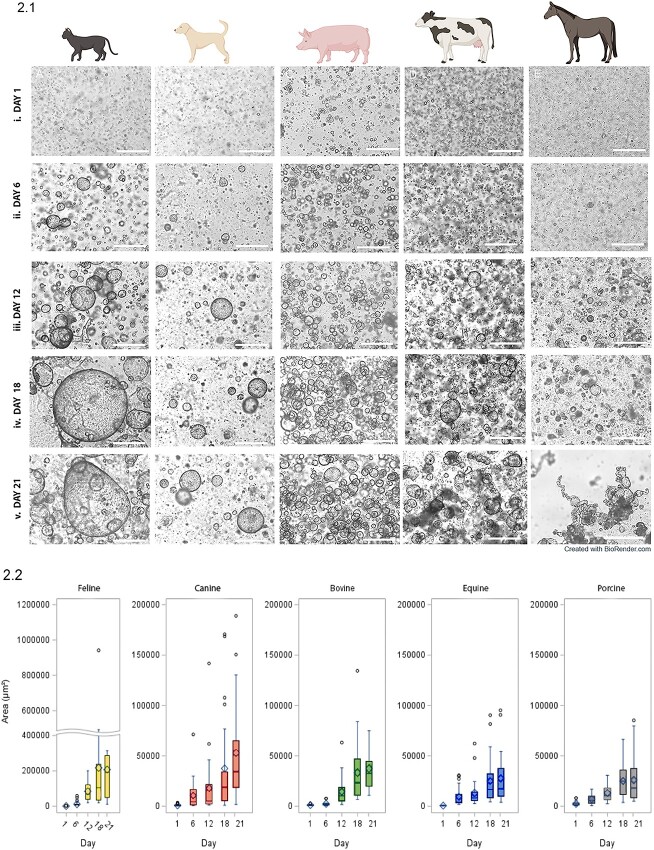
Organoid development from feline, canine, porcine, bovine, and equine OE cells over a period of 21 days in culture. (2.1) Representative bright-field microscopy images showing organoid development from feline (A), canine (B), porcine (C), bovine (D), and equine (E) OE cells at Days 1, 6, 12, 18, and 21 of culture (i, ii, iii, iv, and v, respectively). The scale bars shown on all images correspond to a length of 500 μm. (2.2) Quantification and comparison of organoid sizes for each species, with area growth per day. (2.3) Quantification of the average area (μm^2^) organoid size between species at 1, 6, 12, 18, and 21 days of culture. (2.4) The distribution of organoid sizes over 21 days of culture according to area (μm^2^).

**Figure 2:**
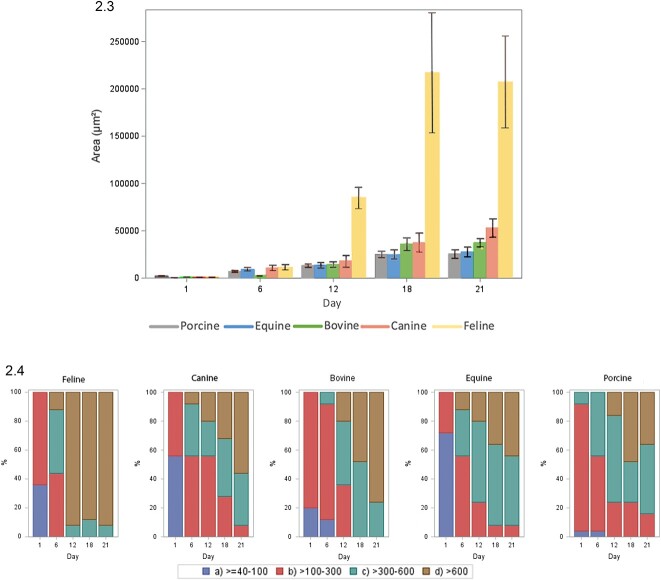
Continue.

### Species variation in organoid phenotype and growth

**Figure 3:**
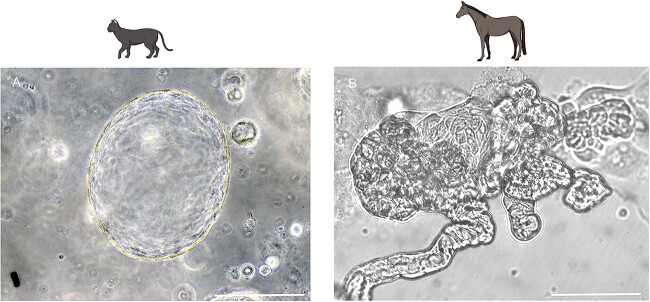
High magnification (×20) brightfield images of feline (A) and equine (B) mature infundibulum organoids at 21-days of culture. The equine organoids started branching as they matured and depicted a more varying and complex shape than the feline OE organoids, which remained more spheroid. The scale bars correspond to a length of 125 μm.

For each species, cultured organoid growth was consistent, but between species, organoid expansion, and size progressed along different trajectories. By comparing the organoid development images taken on the JuLiTM Stage Real-Time Cell History Recorder (NanoEnTek), a marked expansion in size was visually observed for each species from day 6 (Figure 2.1ii). However, feline oviductal organoids had exponential growth in size (perimeter) from day 6 (Figure 2.1ii.A), showing a notably higher rate of expansion from that point compared with other species (Figure 2.1iiB–E). Organoids from all species developed crypts and villi-like structures and internal folding architecture and invaginations, analogous to the mucosal structure of the native oviduct (Figure 2.1v.D and E; 1, 3, 4). Still, phenotypic differences were immediately apparent between the species. To quantify organoid development, individual organoids (*n* = 25) were measured from each sample for each species, and the mean area (μm^2^) was recorded at 1, 6, 12, 18, and 21 days of culture. Variable growth rates of the area (μm^2^) were seen between species for area growth per day (Figure 2.2). Quantifying the average area (μm^2^) organoid size between species illustrated that regardless of area, the oviductal organoid populations developed from single cells and progressively grew in size (Figure 2.3). One-way analysis of variance showed that there were significant differences across time points (days 6, 12, 18, and 21) on the organoid areas of felines *F*(3, 96) = 5.99, *P* < 0.001, canines *F*(3, 96) = 6.14, *P* < 0.001, bovines *F*(3, 96) = 17.06, *P* < 0.001, equines *F*(3, 96) = 5.32, *P* < 0.01, and porcine *F*(3, 96) = 9.42, *P* < 0.001. Overall, Tukey’s multiple comparisons tests showed that in all but one species the mean organoid areas at day 21 were significantly higher than the means at days 6 and 12. In felines, however, the mean organoid area at day 21 was higher than the mean at day 12, but this difference was not statistically significant. Bovine and porcine mean organoid areas at day 18 were also significantly higher than the mean areas at days 6 and 12. Collectively, maximal organoid size growth occurred between days 12 and 18. In addition, this size was compared for each species using area (μm^2^) growth per day, and the distribution of organoid sizes over 21 days of culture was recorded (Figure 2.4). Branching architecture was consistently evident in the equine (Figure 3A), where the oviductal organoids start to branch into luminal structures, reflective of the oviduct, particularly when compared with the rapidly enlarging perimeter observed in the mature feline organoids (Figure 3B). As such, perimeter to area ratios were used measure the complexity index of the 3D biological shapes. When mature organoids (day 21) from each of these species were compared (the higher the value the more complex the shape), equine was the most complex and the feline the least (feline = 0.012, bovine = 0.023, canine = 0.025, porcine = 0.031, equine 0.033).

### Staining of organoid cultures

**Figure 4:**
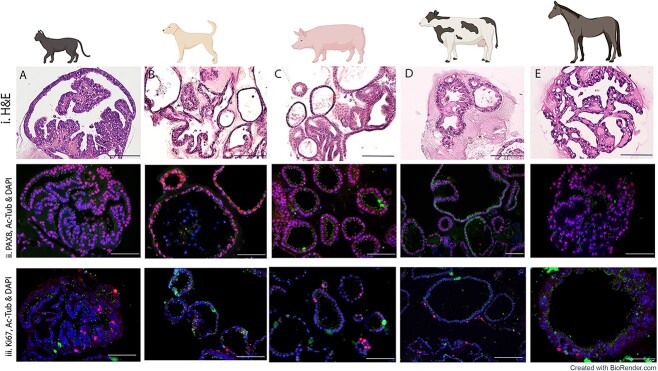
Histological characterization of feline (A), canine (B), porcine (C), bovine (D), and equine (E) oviductal organoids at 21 days of culture. (i) H&E histological staining. H&E shows OE organoids with a monolayer of polarized columnar epithelial cells lining the lumen. In each species, a clear lumen is evident, characterized by the presence of luminal crypts and folds, interspersed with secretory and ciliated cells. (ii) Immunostaining with PAX8 (red), AC-Tub (green), and DAPI (blue) within epithelial cells. Positive staining for AC-Tub indicates ciliated cell differentiation. Positive PAX8 nuclear staining indicates the presence of progenitor secretory cells, which are non-ciliated. DAPI effectively stained the nuclei. Staining patterns mimicked that represented in native oviduct tissue. (iii) Positive nuclear staining for Ki67 (red) indicates cells actively divide as cells outside the replicative cell cycle do not stain positive for Ki67. The staining pattern of Ki67+ cells can be seen consistently but irregularly spaced throughout organoids. AC-Tub+ stained ciliated cells are identified together with DAPI staining cell nuclei. The scale bars shown on all images correspond to a length of 100 μm.

To determine if the organoid cell-network architecture was similar to that observed in the native oviductal epithelium (Figure 4), H&E staining was carried out on oviductal organoid full-thickness sections (Figure 4i) after 21 days of culture. Staining showed that oviductal organoids were composed of a monolayer of polarized columnar epithelial cells, exhibiting equivalent complex internal folding architectural structures. For further comparison, individual cultures were stained with PAX8, AC-Tub, and Ki67, with DAPI serving as a nuclear counterstain after 21 days of culture (Figure 4ii). Mature organoids consistently expressed PAX8+ secretory cells and PAX8-/AC-Tub+ ciliated cells in all species (Figure 4iii). Independent sample *t*-tests showed few statistically significant differences between native and organoid tissues across the different species studied (Supplementary Table S1). However, in canine, the percentages of PAX8, AC-Tub, and Ki67 were significantly higher in organoid tissues. Similarly, compared with native tissue, organoid tissue showed a significantly higher percentage of PAX8 in porcine, of AC-Tub in bovine, and of Ki67 in equine.

### Functional analysis of cilia

**Figure 5:**
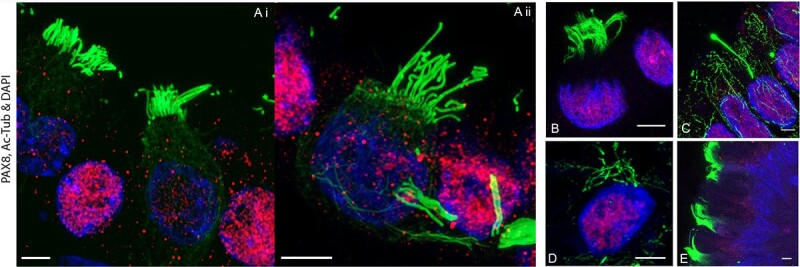
Representative immunofluorescent confocal images showing detailed cilia structures of organoids at 21 days of culture from each species. Ciliated cells contained abundant well-defined cilia, predominantly apically pointed, and anchored to well-aligned basal bodies. Those cells with acetylated microtubules showed positive staining (green) with AC-Tub but were negative for PAX8 secretory cells and vice versa (Ai). In some of these cells, AC-Tub positive cilia can be observed projecting into a different confocal plane of view. (Ai and Aii) porcine, (B) feline, (C) bovine, (D) equine, (E) canine. The scale bars correspond to a length of 4 μm.

Consistent with native oviductal epithelium, AC-Tub+ cilia were identified via IF, and found to be projecting luminally in mature organoids of all species. The presence and detail of AC-Tub+ cells with visible cilia were captured with high magnification using IF confocal imaging (Figure 5), such ciliated cells contained abundant well-defined cilia that were apically pointed and anchored to well-aligned basal bodies. For functional validation of the model, it is established mature organoids should possess a functional ciliary axoneme capable of generating a unidirectional flow and a ciliary beat. As such, motile cilia were observed consistently in the mature organoid population, with mature cilia vigorously motile and recorded using high-speed infra-red differential interference contrast optics. A synchronized, continual beat was recorded in both equine and porcine with actively motile cilia generating a coordinated and continual, unidirectional flow (Supplementary Video S1), of secretion, debris, and medium.

## Discussion

This study aimed to develop and characterize oviductal organoid cultures across multiple domestic species. By developing a standardized model, this study found that mature oviductal organoids, harvested from the distal oviduct, resembled architecturally, structurally, and functionally to their native tissues. In this study, a standardized model was successfully generated. We found that Ki67, a marker of cell proliferation, was observed in all organoid populations, indicating that some cells within the organoids were actively dividing. Furthermore, the progenitor secretory cell marker (PAX8) was also consistently present in mature oviductal organoids in all species investigated. This is particularly pertinent as within oviductal epithelia, the secretory cells (PAX8+) continuously self-renew and give rise to ciliated cells (AC-Tub+) [11]. Hence, organoids recapitulate some of the key features of oviductal tissue regeneration and homeostasis. Lastly, using high-resolution imaging, in real time, ciliated cells were recorded, generating a synchronous and continual beating of motile cilia, which produced a coordinated and unidirectional flow.

### Progenitor cells

In mouse oviducts, a single genetically labeled Pax8+ cell can give rise to a patch of cells containing both secretory and ciliated cells [11]. The ability of Pax8+ cells to self-renew and differentiate is governed by the level of Wnt signaling [11]. PAX8+ cells similarly act as stem/progenitor cells in human fallopian tubes and are the only cells that generate organoids [12]. In this study, we observed that organoids are derived from single non-ciliated cells. The analysis of organoids showed that both ciliated (AC-Tub) and secretory (Pax8) cells are present, suggesting that similar to mouse and humans, PAX8+ progenitor cells give rise to organoids developed from the oviducts of domestic animals.

### Mimicry: in vivo and in vitro differences

Interspersed Ki67+ proliferating cells were detected in the tissue and the resulting organoids, demonstrating the presence of active proliferation in each species. Examination of Ki67 expression pattern across all the species examined in this study showed many expression patterns in oviductal organoids reflective of their native oviductal epithelium. Variations in patterning are consistent with epithelial cell cycle variation, maturity, and likely species-specific differences. Statistical analysis of these results showed that few significant differences existed between native cells and oviducts. Those that did exist are likely to be because of sample deterioration resulting from collecting samples, which are being compared with in vitro produced cell populations. For canines, the maturity level of the animals is likely to also play a factor. Nonetheless, this difference is promising for the model developed; as age, quality of sample, and stages of cycle were circumvented and healthy functional organoid populations generated.

### Species differences in growth

Although mature organoids were observed in all species by 21 days, the interspecies differences in oviductal development rate and structure were not surprising. Many mammalian species display unique features in fertilization physiology and early embryo development. For example, in the bitch, the oocyte is released 2 or 3 days prior to fertilization [7], rather than at the time of ovulation. In the mare, the developing embryo remains in the oviduct for 5 days after fertilization [27, 28], contrasting markedly with the cow and sow, in which the embryo/s remain in the oviduct for only 3 and 2 days, respectively [29, 30]. In addition, litter size should be considered when analyzing the oviduct’s function; the sow can carry 16 piglets per gestation, whereas, in the mare, only one offspring is typically carried to term. Such functional idiosyncrasies must be considered when comparing oviductal epithelium. In this study, the comparative differences in organoid size (seen in the feline) and complexity observed (in the equine) may indeed relate to the different patterns of early embryonic development between species. The differences in growth trajectories and proliferation rates of the organoids at different time points during in vitro culture may reflect the species-specific differences in the oviductal tube physiology [31].

### Functional cilia

Ciliated cells are essential for the normal functioning of the oviducts. Similar to the native tissue, we found mature, functionally motile ciliated axonemes in organoid populations. Initially, mature ciliated axonemes were identified on each organoid by the presence of AC-Tub (Figure 5). Confocal microscopy imaging of cilia sections confirmed the detail and structural complexity that each mature axoneme possessed and in some cells, cilia started basally and projected off the 5 μm thickness plane of the section, suggesting a mature and differentiated state of ciliated cells. Further statistical analysis of ciliated cells illustrated that few significant differences existed between populations. However, only when the organoids were also visualized and recorded in real time, under high resolution, the proportion/distribution of ciliated cells more closely represented native epithelia. Thus, it is presumed some structural clarity of the small delicate spheroid organoid is lost when sectioned, imaged, and visualized two dimensionally. Excitingly, by visualizing these live population, in real time, under high resolution, the aforementioned cilia were observed producing a synchronous and continual beat frequency resulting in a unidirectional flow. As the mammalian oviduct is one of the organs that relies heavily on the motility of cilia for its normal functions [32], the identification of actively organized and motile cilia supports the functionality of the organoid model described in this manuscript.

### Commercial and veterinary advancement

Knowledge gained from such cross-species comparisons highlights the diversity that exists [33]. In a commercial setting, reduced fertility rates may be because of the lack of species-specific knowledge. Where, poor-fertility or subfertility often translates to financial losses for producers and compromised welfare arising from repeated invasive veterinary interventions [34]. So, although IVF techniques have progressed over the last 40 years, and are now used in multiple domestic and non-domestic species around the world [35], a number of problems remain unsolved or conditions not yet optimized [36]. IVF in the bovine species is of particular interest, with its commercial scale now comparable to human IVF [35]. Likewise, for the equine, however, intra-cytoplasmic sperm injection is currently the only commercial method for producing equine embryos in vitro. Research in the equine has recently focused on the early secretions of both the embryo and the maternal host [37–40] and highlighted the need for a more profound study of the oviduct. As species-specific differences between mammals are notable and varied, the traditional medical model, beginning on laboratory rodents before clinical translation to humans [41] or mammals, is less than ideal. On these grounds, individualized organoid models derived from farm and companion animals have great potential to contribute to veterinary medicine and the health of the human population. The insights gained may benefit human fertility treatments and wildlife conservation efforts.

### Limitations

Anatomically, in human and mouse native oviduct, the proportion of ciliated cells is most numerous in the distal oviduct [11]. Since fimbrial cells have been shown to possess the highest organoid-forming capacity [12, 16, 17], this anatomical region was harvested for this study. Previously, organoids provided a challenge because of the apical, or luminal, surface of the epithelium (where the cilia are located) being enclosed within the organoid interior. However, several techniques are now available to overcome past difficulties, including micro-injection via micromanipulation [42, 43], flipping the organoids inside out using an apical-out reversal protocol [44] or vigorous pipetting [45] to allow apical exposure. This latter technique is applicable for shorter-term experiments, and is the preferred technique used in this protocol. As previously mentioned, the collection of oviductal specimens was limited to those discarded samples and the cyclicity, precise age, and exact health status could not always be accounted for in the present study. Yet, the findings seem to provide a framework for robust models in species other than human or mouse.

## Conclusion

This study has succeeded in defining and characterizing the growth and differentiation of oviductal organoids in domestic species and, in so doing, has demonstrated the effectiveness of a methodology that can be applied to future research on the reproductive biology of key livestock species. The development of an oviductal organoid model for domestic animal species allows direct access to the environment in which conception and early embryonic development occur. As such, it creates a platform for investigating the critical attributes of a biologically supportive oviductal environment, which may include, but is not limited to further investigations into oviductal secretion, oviductal biomechanics, and the influence of the oviductal milieu on both embryo and spermatozoa. Such models may play a crucial role in addressing key issues relating to the efficacy of assisted reproductive technologies procedures, advances in animal welfare, and the preservation of diversity. In addition, the species-specific variation in cilia morphology and beat frequency would be of interest. Given that the galine and the ovine models are frequently used in female reproductive and pregnancy studies, including both of these additional species in future organoid models would be of value. Lastly, future research could include single-cell sequencing and proteomic analysis of both epithelium and secretome. Research comparing organoid and native tissue in this way could assist us in better understanding the species-specific molecular signaling required for fertilization and embryo development. Developing a clearer understanding of the organoid molecular signaling cascade could transition into a more accurate model for in vitro fertilization. As the oviduct is the site of sperm capacitation, fertilization, and early embryonic life, the findings of this study open up the potential to use this organoid model to achieve fundamental insights into the understanding of processes that are critical for the reproductive process both in vivo and in vitro.

## Author contribution

EL, ZG, RJA, PT Designed research; EL, AG, VB, HD Performed research; HD, RL, MB Contributed new reagents; EL, AG, VB, HD Analyzed data; EL, MB, ZG, RJA, PT Wrote the paper.

## Supplementary Material

Supp_TABLE_1_IF_cell_statistics_ioad030

S1_video_Cilia_beating_ioad030

## Data Availability

The data underlying this article are available in the article and in its online supplementary material.
