# Supplementary material for: Establishment and characterization of oviductal organoids from farm and companion animals
Source: Biol Reprod. 2023 Mar 14;108(6):854–65. doi: 10.1093/biolre/ioad030 (PMC10266943; doi:10.1093/biolre/ioad030)
Supplement: Supp_TABLE_1_IF_cell_statistics_ioad030 [file supp_table_1_if_cell_statistics_ioad030.docx]

|  |  | Group of native tissue | |  | Group of organoid tissue | |  |  |  |
| --- | --- | --- | --- | --- | --- | --- | --- | --- | --- |
| Species |  | Mean | (SD) |  | Mean | (SD) |  | t-test | p-value |
|  |  |  |  |  |  |  |  |  |  |
|  |  | PAX8 | | | | | | | |
| Feline |  | 73.9 | (21.8) |  | 85.1 | (7.6) |  | 0.84 | .4492 |
| Canine |  | 61.8 | (2.7) |  | 81.0 | (7.2) |  | 4.30 | **.0127** |
| Equine |  | 68.0 | (10.5) |  | 62.7 | (9.7) |  | 0.64 | .5563 |
| Bovine |  | 79.0 | (12.1) |  | 93.9 | (4.2) |  | 2.01 | .1142 |
| Porcine |  | 28.9 | (4.9) |  | 79.0 | (16.4) |  | 5.06 | **.0072** |
|  |  |  |  |  |  |  |  |  |  |
|  |  | Ac-Tub | | | | | | | |
| Feline |  | 18.9 | (5.8) |  | 42.2 | (14.7) |  | 2.56 | .0623 |
| Canine |  | 15.0 | (3.1) |  | 49.5 | (8.4) |  | 6.69 | **.0026** |
| Equine |  | 21.1 | (1.6) |  | 29.7 | (14.9) |  | 1.01 | .3717 |
| Bovine |  | 28.3 | (5.6) |  | 46.7 | (7.0) |  | 3.55 | **.0237** |
| Porcine |  | 18.3 | (2.9) |  | 33.3 | (27.4) |  | 0.94 | .4009 |
|  |  |  |  |  |  |  |  |  |  |
|  |  | KI | | | | | | | |
| Feline |  | 5.4 | (0.7) |  | 7.0 | (4.6) |  | 0.57 | .5968 |
| Canine |  | 3.8 | (2.1) |  | 11.6 | (2.7) |  | 3.95 | **.0168** |
| Equine |  | 8.1 | (1.8) |  | 3.7 | (1.4) |  | 3.34 | **.0289** |
| Bovine |  | 2.4 | (0.6) |  | 11.3 | (5.7) |  | 2.71 | .0538 |
| Porcine |  | 6.5 | (3.0) |  | 6.5 | (5.5) |  | 0.03 | .9795 |

**Table 1.** Comparison of PAX8, Ac-Tub and Ki67 in Native and Organoid tissue across all species.
